# Association of the methylene-tetrahydrofolate reductase gene rs1801133 C677T variant with serum homocysteine levels, and the severity of coronary artery disease

**DOI:** 10.1038/s41598-020-66937-3

**Published:** 2020-06-22

**Authors:** Nadia Bouzidi, Majed Hassine, Hajer Fodha, Mejdi Ben Messaoud, Faouzi Maatouk, Habib Gamra, Salima Ferchichi

**Affiliations:** 1 University of Monastir, Faculty of Pharmacy, Clinical and Molecular Biology Unit, UR 17ES29, 5000 Monastir, Tunisia; 2 University of Monastir, Cardiology A Department Fattouma Bourguiba University Hospital, Cardiothrombosis Research Laboratory, LR12SP16, 5000 Monastir, Tunisia; 3 University of Monastir, Faculty of Pharmacy, Biochemistry and Molecular Biology Laboratory, Monastir, Tunisia; 4 University of Monastir, Cardiology B Department Fattouma Bourguiba University Hospital, Cardiothrombosis Research Laboratory, LR12SP16, 5000 Monastir, Tunisia

**Keywords:** DNA, Interventional cardiology, Cardiovascular genetics, Biomarkers

## Abstract

This study aimed to investigate whether the single nucleotide polymorphism C677T (rs1801133) of the methylene-tetrahydrofolate reductase (MTHFR) gene was associated with the risk of coronary artery disease (CAD) and circulating homocysteine (Hcy) levels in Tunisian population. 310 angiografically diagnosed CAD patients and 210 controls were enrolled in this study. The MTHFR C677T (rs1801133) polymorphism was genotyped, and the Hcy concentrations were measured. The severity of CAD was evaluated using the Gensini scoring system. Compared to the CC genotype, the TT genotype confers a higher risk for CAD severity with an OR = 9.07 and 95% CI = 3.78–21.8. The T allele was the predisposing allele for CAD and that it was probably associated with CAD severity. The area under the ROC curve for Hcy was 0.764 (95% CI 0.660 to 0.868, p = 0.001). The receiver operating characteristics curve (ROC) for Hcy showed its useful prediction of CAD. Hcy levels were not significantly associated with CAD severity expressed by Gensini Score (GS). The MTHFR C677T (rs1801133) polymorphism influences circulating Hcy levels. The MTHFR C677T polymorphism and hyperhomocysteinemia could have an important role in the prediction of the presence and not the severity expressed by GS of CAD.

## Introduction

Homocysteine (Hcy) was known to be an independent risk factor for cardiovascular disease observed even in very young patients with extremely high serum Hcy concentrations. Previous studies have shown that Hcy can be associated with oxidative stress, activate platelet aggregation and cause endothelial dysfunction and proliferation of vascular smooth muscular cells, which induces the appearance of vascular lesions^1^. Hyperhomocysteinemia (HHcy) in elderly can be due to deficiency of enzymes involved in Hcy and B vitamins metabolism, nutritional impairements in vitamin cofactors, age, drugs use or other factors as lifestyle conditions^2,3^. It has been hypothesised that Hcy alterates lipid metabolism. Several studies showed that Hcy affects high density lipoprotein cholesterol (HDL-C) blood levels via inhibiting apolipoprotein (ApoA)-I synthesis and increase HDL-C liberation^4^. Hcy metabolism involves two major pathways: (1) the remethylation pathway and (2) the transsulfuration pathway. In the remethylation pathway, once the folate is formed in the blood, it provides a methyl group to Hcy in the presence of vitamin B12-dependent enzyme methionine synthase leading to the formation of tetrahydrofolate. Hcy is subsequently converted to methionine. The transsulfuration pathway consists in the reaction of Hcy with serine catalysed by cystathionine b-synthase using vitamin B6 as a cofactor. This reaction leads to the formation of Cystathionine which is then converted into cysteine. Cysteine is converted into sulfates^5^. Methylene tetrahydrofolatereductase (MTHFR) is the enzyme involved in the metabolism of methionine producing Hcy, the sulfur amino acid^1^. Several mutations in the MTHFR gene are involved in the alteration of MTHFR activity. Among these polymorphisms, the variant of the MTHFR gene at position 677 from C to T (alanine replaced by valine) is widely regarded as the main cause of HHcy^6^. Previous studies have shown that TT variants of MTHFR C677T polymorphism have been associated with elevated serum Hcy concentrations and severity of coronary lesions suggesting its important role as coronary artery disease (CAD) marker^7^. It has been suggested that the rs1801133 polymorphism could affect the methylation state of DNA and thus alterates lipid metabolism which is involved in CAD process^8^. Based in its central role, various diseases are associated with MTHFR polymorphism as neural tube failure, Alzheimer’s and brain diseases and severe vascular disease^2^. Although several studies have suggested that HHcy can cause CAD, the association between Hcy and the severity of coronary lesions, particularly in patients with acute coronary syndrome (ACS), has rarely been reported. It has been suggested that MTHFR C677T gene polymorphism may influence the association between Hcy levels and CAD occurrence risk^7^. The purpose of this study was to test the hypothesis that the MTHFR polymorphism C677T influences Hcy concentrations. In addition, as secondary objective we also investigated the association between severity of CAD, MTHFR C677T polymorphism and its serum levels in a Tunisian population.

## Results

### Baseline characteristics of CAD patients and controls

The baseline characteristics of the study subjects are shown in Table 1. The majority of CAD patients were smokers and males. The mean of age among patients was higher than that of controls (60.3 ± 11.0 *vs*. 33.2 ± 12.2 (years)).

**Table 1:** Baseline characteristics of the study subjects.

Characteristics	Controls N = 207	Patients N = 310	p
Age, years	33.2 ± 12.2	60.3 ± 11.0	<0.001
Male, n (%)	61(29.5)	232(74.8)	<0.001
BMI, Kg/m²	25.1 ± 3.6	27.5 ± 4.3	<0.001
Smoker, %	8.2	41.0	<0.001
Hypertension, %	0	47.1	<0.001
Diabetes, %	0	48.4	<0.001
Dyslipidemia, %	0	23.5	<0.001

BMI: body mass index, CAD: coronary artery diseases. Data are expressed as mean ± SD or n (%). P < 0.05.

### Distribution and association of rs1801133 C677T of MTHFR gene with CAD

The highest levels of Hcy were schown in TT genotype patients compared to CT and CC genotypes (32.6 (17.7–50.0) *vs*. 17.9(9.5–50.0) *vs*. 15.1(7.9–40.3) μmol/L). All TT genotype patients had high levels of Hcy (≥ 15 µmol/L). 75.0% of TT genotype patients had a GS ≥ 40. The Highest levels of GS were schown in TT genotype patients compared to CT and CC genotypes (61.4 ± 42.6) *vs*. 46.0 ± 37.0 *vs*. 49.7 ± 44.6). TT genotype carriers have higher lipid concentrations but not significant (Table 2).

**Table 2:** Clinical and biochemical characteristics of patients group.

Characteristics	Patients			
	CC	CT	TT	P
Age (y)	56.8 ± 11.2	59.0 ± 9.0	64.5 ± 2.1	0.081
Males %	43.1	37.5	10.4	0.573
BMI (Kg/m²)	26.6 ± 3.3	27.4 ± 3.8	27.1 ± 2.8	0.790
Stenosis (%)				
[50–70] % >70%	33.3	28.6	15.4	0.562
	66.7	71.4	84.6	
Vessel Disease Number				
One Vessel Multi Vessel	43.3	63.2	36.8	0.793
	56.7	61.4	38.6	
Gensini score groups				0.147
GS < 40	46.9	55.2	25.0	
GS > = 40	53.1	44.8	75.0	
Homocysteine (µmol/L)				0.033
Hcy < 15	47.1	41.7	0	
Hcy > =15	52.9	58.3	100	
TG (mg/dL)	1.2 (0.27–4.4)	1.4 (0.5–6.1)	1.5 (0.5–4.8)	0.732
TC (mg/dL)	3.8 (0.9–6.5)	4.2 (1.8–7.3)	4.5 (2.0–6.0)	0.287
LDLc (mg/dL)	2.3 (0.1–4.8)	2.5 (0.9–5.2)	2.8 (0.4–4.3)	0.493
HDLc (mg/dL)	0.8 (0.2–2.3)	1.0 (0.3–1.2)	1.1 (0.7–1.9)	0.024
ApoA1 (mg/dL)	93.6 ± 27.3	91.2 ± 29.2	101.2 ± 16.4	0.562
ApoB (mg/dL)	76.9 ± 26.5	77.1 ± 24.8	84.2 ± 30.0	0.671
Lp(a) (mg/dL)	11.1 (2.3–81.0)	9.5 (2.3–62.0)	12.5 (2.3–93.2)	0.977
TC/HDLc	4.7 ± 1.3	4.8 ± 1.7	4.2 ± 1.4	0.460
LDL/HDLc	2.9 ± 1.3	2.9 ± 1.7	1.3 ± 0.6	0.749
TG/HDLc	1.7 ± 1.1	2.0 ± 1.7	1.3 ± 0.6	0.261
HDLc/ApoA1	0.9 (0.3–2.4)	1.1 (0.5–1.8)	1.2 (0.8–1.7)	0.193
HDLc/Lp(a)	6.5–1.7–44.4)	7.0 (2.2–36.0)	11.0 (1.6–47.4)	0.705
AIP	0.2 ± 0.2	0.2 ± 0.3	0.1 ± 0.3	0.225
ApoB/ApoA1 (10^-2^)	95.5 ± 55.0	98.7 ± 91.2	86.0 ± 34.6	0.856
HsCRP (mg/L)	5.7 (0.2–190.0)	5.9 (0.2–204.0)	5.8 (0.2–1020.0)	0.785
Hcy (µmol/L)	15.1 (7.9–40.3)	17.8 (9.5–50.0)	32.6 (17.7–50.0)	0.009
GS	40.0 (2.0–192.0)	32.0 (2–120)	40.0 (6.0–156.0)	0.419

ApoA-1: apolipoprotein A-1, ApoB: apolipoprotein B, BMI: body mass index, HDL-c: high density lipoprotein cholesterol, Hs-CRP: high sensitivity C-reactive protein, Hcy: homocysteine: GS: Gensini score, IL-6: interleukin 6, LDL-c: low density lipoprotein cholesterol, Lp(a): lipoprotein(a). TC: total cholesterol, TG: triglyceride. Values are expressed as the mean ± SD or median (Min - Max). Statistical significance P < 0.05.

### Distribution of biochemical characteristics in CAD patients across MTHFR C677T genotypes

Regarding genotypic distribution, both cases and controls were in Hardy-Weinberg equilibrium for the two polymorphisms. Compared with controls, the CAD subjects had CT and TT genotypes more frequently and had CC genotype less frequently (38.9% *vs*. 37.8%, 18.9% *vs*. 2.7%, 42.2% *vs*. 59.5%, respectively, P = 0.039. The OR for CAD was 1.5 (95% confidence interval (CI), 1.0–2.1; P = 0.050) of the CT genotype and 9.1 (95% CI, 3.8–21.8; P < 0.001) of the TT genotype. Interestingly, CAD patients carrying minor T allele were significantly associated with a higher risk of CAD compared with those carrying the C allele (OR = 2.2, 95% CI = 1.7–2.9, P < 0.001) (Table 3).

**Table 3:** Genotypic and allelic distribution and association of rs1801133 polymorphism of MTHFR gene with CAD.

MTHFR C677T	Controls N = 207	CAD N = 310	P	OR (%)	CI 95%	P
**Genotype**						
CC CT TT	123 (59.5)	131 (42.2)	0.039	1	—	—
	78 (37.8)	121 (38.9)		1.457	0.999–2.122	0.050
	6 (2.7)	58 (18.9)		9.07	3.78–21.8	<0.001
**Allele**						
C T	324 (78.4)	383 (61.7)	<0.001	1	—	—
	90 (21.6)	237 (38.3)		2.227	1.7–2.9	<0.001

### ROC Curve for homocysteine in predicting CAD

**Figure 1:**
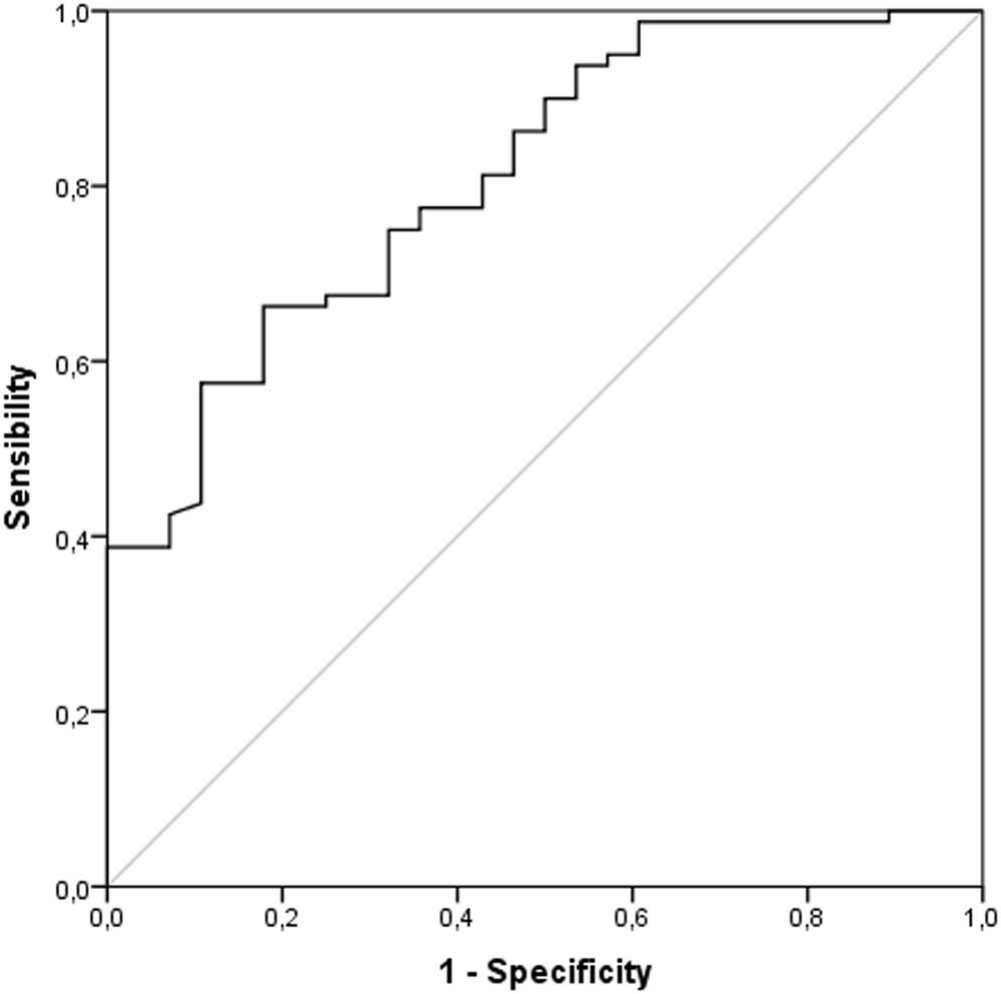
ROC analysis for Hcy levels in predicting CAD. Area under the ROC curve for Hcy was 0.764 (95% CI 0.660 to 0.868, p = 0.001).

Plasma Hcy levels were significantly higher in patients with CAD than controls (18.6(3.7–50.0) µmol/L and 9.9(0.2–22.4) µmol/L, respectively; P < 0.01). The receiver operating characteristic curve (ROC) for Hcy as an indicator of the presence of CAD is shown in Fig. 1. The area under the ROC curve (AUC) for Hcy was 0.764 (95% CI 0.660 to 0.868, p = 0.001).

### Association between homocysteine levels and Gensini score

Based on the median of GS, CAD subjects were separately divided into two groups, low GS (<40), and high GS (≥40). As shown in Table 4, the Hcy levels were higher in the high GS group and p value tended to be significant (p = 0.075).

**Table 4:** The homocysteine levels according to Gensini score groups.

GS	Hcy (µmol/L)	P
<40	14.2 (3.7–50.0)	0.075
≥40	21.1 (7.9–50.0)	

Hcy: homocysteine; GS: gensini score. Statistical significance P < 0.05.

## Discussion

The main findings in this study were the elevation of Hcy levels influenced by the C677T polymorphism of the MTHFR gene in CAD patients. Hcy levels, as shown in ROC curve, could be a useful tool in the prediction of CAD. The frequency of CT and TT genotypes and T allele of rs1801133 were higher in the CAD patients compared with controls. These results suggest that the C677T polymorphism could be associated with increased susceptibility to develop CAD in Tunisian population. In contrast, high levels of Hcy and C677T polymorphism frequencies of the MTHFR gene in CAD patients were not associated with the severity of CAD expressed by GS.

The C677T polymorphism of the MTHFR gene related to Hcy metabolism has been studied in several cardiovascular pathologies, diabetes and other inflammatory diseases. This polymorphism consists of a point mutation where the cytosine is replaced by thymine or a substitution of alanine by valine at the protein position A222V or at the allelic position C677T^9^. The study of this polymorphism revealed significant association between patients and controls in genotype frequencies. According to previous studies results, the distribution of C677T polymorphism appears to be very heterogeneous and genotype frequencies differ from one study to another. In particular, a Turkish team showed a genotypic distribution CC: CT: TT = 40.0%: 47.3%: 12.7% in 243 coronary patients. In a Chinese study, the genotypic distribution in 106 patients was CC: CT: TT = 63.8%: 25.7%: 10.5%^10^. In this study the genotypic distribution in patients was respectively CC: CT: TT = 42.2%: 38.9%: 18.9% which was similar to previous studies. Several studies have not found an association between the mutated gene C677T of MTHFR and the subsequent development of cardiovascular diseases^10^. The frequency of TT genotype in healthy subjects varies by 1.4% in Africa and America, 5.1% in Germany, 10% in Japan and Australia^11^. A Tunisian study of 185 apparently healthy subjects in 2005 showed an allele frequency of 17.8% and a genotypic frequency of 5.4%^12^.

Given the obvious metabolic link between high levels of Hcy and the MTHFR enzyme observed in our patients, we investigated the influence of MTHFR gene C677T polymorphism on plasma concentrations of Hcy. Our results were consistent with some studies and disagreed with others. We found significant association between Hcy levels and the generated genotype (TT) and (T) allele of the MTHFR C677T gene polymorphism. Previous Tunisian studies, as those of Kerkeni M. *et al*. conducted in 2006^13^ and Ghazouani L. *et al*. in 2009^14^ showed that the C677T polymorphism of MTHFR was associated with cardiovascular disease and with elevated Hcy levels. These and other previous studies have suggested that this polymorphism could be considered as a potential genetic cardiovascular risk factor for HHcy^15^. Thermolability of the MTHFR induces an elevation of Hcy concentrations by the inactivation of the resulting active dimer adversely affecting the binding of the flavin adenine dinucleotide^16^ which alterates Hcy methylation pathway and MTHFR activity^17^. It has been suggested that the 677TT polymorphism of the MTHFR gene has been associated with endothelial dysfunction and vascular oxidative stress due to high concentrations of 5-methyltetrahydrofolate (5-MTHF). In other studies, MTHFR-linked hyperhomocysteinemia has been well known as the leading cause of endothelial dysfunction and atherogenesis. The variant MTHFR 677TT influences the levels of 5-MTHF more clearly than Hcy levels^18^.

Compared with the CC genotype, the TT genotype confers a higher risk for CAD severity with an OR = 9.07 and 95% CI = 3.78–21.8. This confirmed that the T allele was the predisposing allele for CAD and that it was probably associated with CAD severity. Previous studies showed that the T allele of the rs1801133 polymorphism was associated with an increased CAD susceptibility and elevated levels of TG, TC and LDL-C, and reduced levels of HDL-C. It has been suggested that the resulting dyslipidemia might be one of the important reasons in the progression of CAD^8^. Another studies reported that the coronary artery patients with MTHFR 677 T allele had decreased levels of glutathione and catalase activity and as a result antioxidant capacity reduction^19^.

Even though the highest levels of Hcy were shown in the group of GS > 40, we didn’t find a significant association between high levels of Hcy and severity of CAD (p = 0.075). The lack of association between Hcy levels and MTHFR C677T polymorphism with the GS and the heterogeneity of genotype frequencies of the MTHFR C677T gene polymorphism may be due to the multifactorial nature of CAD and the very likely existence of interactions between HHcy and other cardiovascular risk factors. Nutritional intake of folate, deficiency of vitamin B12 and B6 could be factors influencing Hcy concentrations given their contribution as co-factors in the control of its metabolism as well as the presence of other mutations and the action of conventional cardiovascular risk factors such as hypertension and smoking, which may affect Hcy concentrations^15^.

The association between HHcy, MTHFR C677T polymorphism and the severity of CAD has been poorly studied. A significant association between high levels of Hcy and the severity of CAD, expressed by the GS independently of MTHFR polymorphism, was observed in Rassoul F. *et al*. findings^20^. Another study showed that high blood levels of Hcy were related to the severity of CAD, as assessed by the atheroma burden index^21^. Current evidence suggests that high levels of Hcy may aggravate CAD via its interaction with both the endothelial cells and cardiomyocytes. A previous study showed that a TT genotype of MTHFR C677T gene polymorphism was associated with higher incidence rate of vulnerable plaque and high Hcy level, which were both considered as predictors of the atherosclerotic plaque instability^22^. A previous study conducted in a Chinese Han population revealed that the MTHFR C667T is associated with the risk of CAD and the TT genotype in MTHFR C667T may increase the severity of CAD as defined by a GS ≥ 22^23^. It has been reported that the rs1801133 polymorphism influences the Hcy blood levels in various populations such as Americans, Africans, Asians, Turkish and Brazilians^8^. In North American Caucasian population, the TT genotype was found to have effect on Hcy levels, and was no associated with CAD^24^. Other studies of some populations from Ireland, The Netherlands and Japan showed an association between the C677T variant and both with CAD, and with early onset of CAD^25^.

Some limitations of our study need to be considered while interpreting the results. Firstly, we did not perform some statistical analysis as multivariate linear analysis of the association between the high levels of Hcy (>15 µmol/L) and other biochemical and clinical parameters. Secondly, our study design does not consider the association between Hcy levels and nutritional factors as circulating levels of folate, vitamin B12 and B6. Among several polymorphisms involved in the metabolism of Hcy, only one SNP site was focused in the present study. The presence of other mutations and the action of conventional cardiovascular risk factors could influence Hcy concentrations. Future studies will need to assess the association of hyperhomocysteinemia, with the other polymorphisms of its metabolism as the A1298C variant. Thirdly, the controls included in this study were blood donors and didn’t undergo coronary angiography, controls and cases vary in age and sex which may be a selection bias. Fourthly and lastly, the relevance of homocysteine levels as a prediction tool of CAD was stated by the only use of ROC curve. Other statistical analysis as multivariate regression analysis could be done to validate this and could be at least mentioned in the discussion.

In conclusion, circulating levels of Hcy were influenced by MTHFR C677T polymorphism and highest levels were associated with the TT genotype. The results revealed that MTHFR C667T polymorphism and HHcy have an important role in the prediction of CAD, but no significant association between Hcy levels and MTHFR C677T polymorphism and severity of CAD expressed by GS was shown.

## Methods

### Subjects

A total of 310 subjects who were recruited for suspected or known coronary atherosclerosis at the department of cardiology in Fattouma Bourguiba Hospital were affiliated to Monastir University. Patients who had renal dysfunction, inflammatory, autoimmune or malignancy disease and renal dysfunction were excluded. Thus, 207 blood donors subjects recruited in the blood bank department in the Tahar Sfar University Hospital of Mahdia were enrolled as controls in this study. This protocol was approved by the Research Ethics Committee of Farhat Hached University Hospital. All research was performed in accordance with relevant guidelines and regulations. Informed consent was obtained from all participants.

### Serum Homocysteine measurement

Serum Hcy levels were measured by Fluorescence Polarization Immunoassay method using an analyzer (AXSYM ABBOTT, Germany). HHcy is defined as serum Hcy levels above 15 µmol/L.

### Lipid profile test

Total cholesterol (TC) and triglyceride (TG) and HDL-C were measured by enzymatic colorimetric method, light density lipoprotein cholesterol (LDL-C) was estimated by the Friedewald equation. ApoB, apoA-1, and Lipoprotein (a) (Lp(a)) measurements were carried out by means of the turbidimetric method (Cobas Integra 600, Roche).

### Angiographic severity

CAD was defined as>50% luminal narrowing of at least one major epicardial vessel. CAD severity was ascertained by assessing the multivessel disease extent [one-, two-, or three-vessel disease (>50% stenosis)]. The degree of coronary stenosis was classified as moderate (50–70% stenosis) and severe (>70% stenosis) according to previously published guidelines^26^. The severity of CAD was determined by Gensini scoring system which is defined as follow^27^: briefly, if any branch of main coronary artery Left Main Artery (LM), LAD, Left Circumflex Coronary Artery (LCX) and RCA has stenosis reaching 1–24% of the internal lumen diameter, 1 point is given. Similarly, 2 is given for 25–49% stenosis, 4 for 50–74%, 8 for 75–90%, 16 for 91–99% and 32 for 100% or total occlusion. Depending on the lesion location, the single lesion score and the coefficient, the final Gensini total score was calculated.

### Genomic DNA extraction

Genomic DNA was extracted from blood leukocytes collected into tubes containing ethylene diamine tetra acetic acid (EDTA) by salting out method. The isolated DNA was also stored at −80 °C.

### Genomic DNA purity measurement

The purity and the concentration of isolated DNA was measured using Nano2000 spectrophotometry (Thermoscientific, Waltham, MA, USA) and genomic DNA samples with concentration >200 ng/μL and purity levels 1.7^28^.

### Genotyping

**Figure 2:**
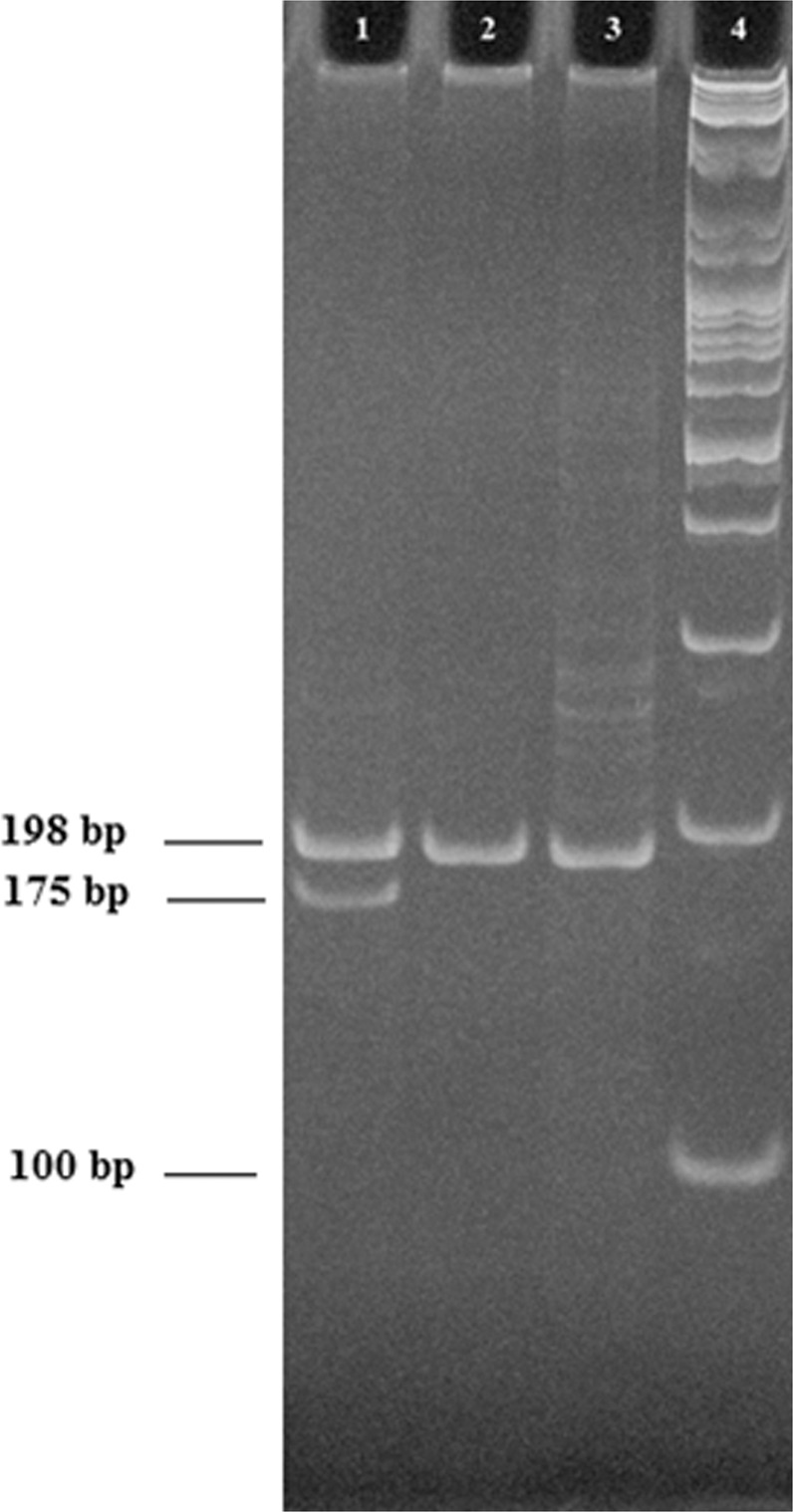
Genotypes of MTHFR C677T polymorphism on 10% polyacrylamide gel electrophoresis showing PCR-RFLP products. L1: CT genotype; L2: CC genotype; L3: undigested PCR product; L4: DNA standard marker (100 bp).

MTHFR C677T (rs1801133) genotyping was performed using polymerase chain reaction (PCR) restriction fragment length polymorphism (RFLP). The PCR primers sequence was: forward, 5′ TGA AGG AGA TGT CTG CGG GA-3′, reverse, 5′AGG ACG GTG CGG TGA GAG GTG-3′. Amplification was performed on 1 µL of genomic DNA. Reaction mixtures also used 2.5 µL of 10X PCR buffer, 0.4 µL of each primer, 4 µL of deoxy nucleotides (dNTPs) mix and 0.2 µL of Taq polymerase. The cycling profile was 95 °C for 5 min, followed by 35 cycles at 95 °C for 1 min, 59 °C for 1 min, 72 °C for 2 min, and 72 °C for 7 min. The amplified DNA fragments (198 bp) were evaluated after their digesting overnight at 37 °C by a restriction enzyme HinfI. Digestion of DNA fragments (10 µL) was mixed with 4 µL of loading buffer in each well and separated on 10% acrylamide- bis acrylamide gels (19:1) 40% (Promega) for 3 h at 30 V in Tris- borate- EDTA buffer (10×) and stained with ethidium bromide (1 mg/L). After digestion, the fragment lengths were 198 bp for the CC genotype; 198, 175, and 23 bp for the CT genotype; and 175 and 23 bp for the TT genotype (Fig. 2).

### Statistical analyses

Statistical Package for Social Sciences version 23.0 was used for data analysis. Normally distributed data were expressed as mean ± standard deviation (SD), and comparisons between patients were performed using Student’s t test. Non-normally distributed data were expressed as medians (minimum value – maximum value), and comparisons between patients were performed using the Mann–Whitney U test. Categorical data were expressed as percentages, and comparisons between patients were performed using chi-square test. Kruskal–Wallis tests or one-way analysis of variance tests were used to compare multiple groups^8^. Genotype/allele frequencies and difference in genetic and allelic frequencies considering Hardy–Weinberg Equilibrium were determined by chi-square analysis. P ≤ 0.05 was considered statistically significant. Odds ratio (OR) with 95% confidence interval (95% CI) was calculated by an online statistical calculator available at www.medcalc.net.
